# Lumbosacral Echinococcosis

**DOI:** 10.4269/ajtmh.22-0564

**Published:** 2023-02-06

**Authors:** Shutao Gao, Yukun Hu, Weibin Sheng

**Affiliations:** Department of Spine Surgery, Xinjiang Medical University Affiliated First Hospital, Urumqi, China

**Figure 1.**
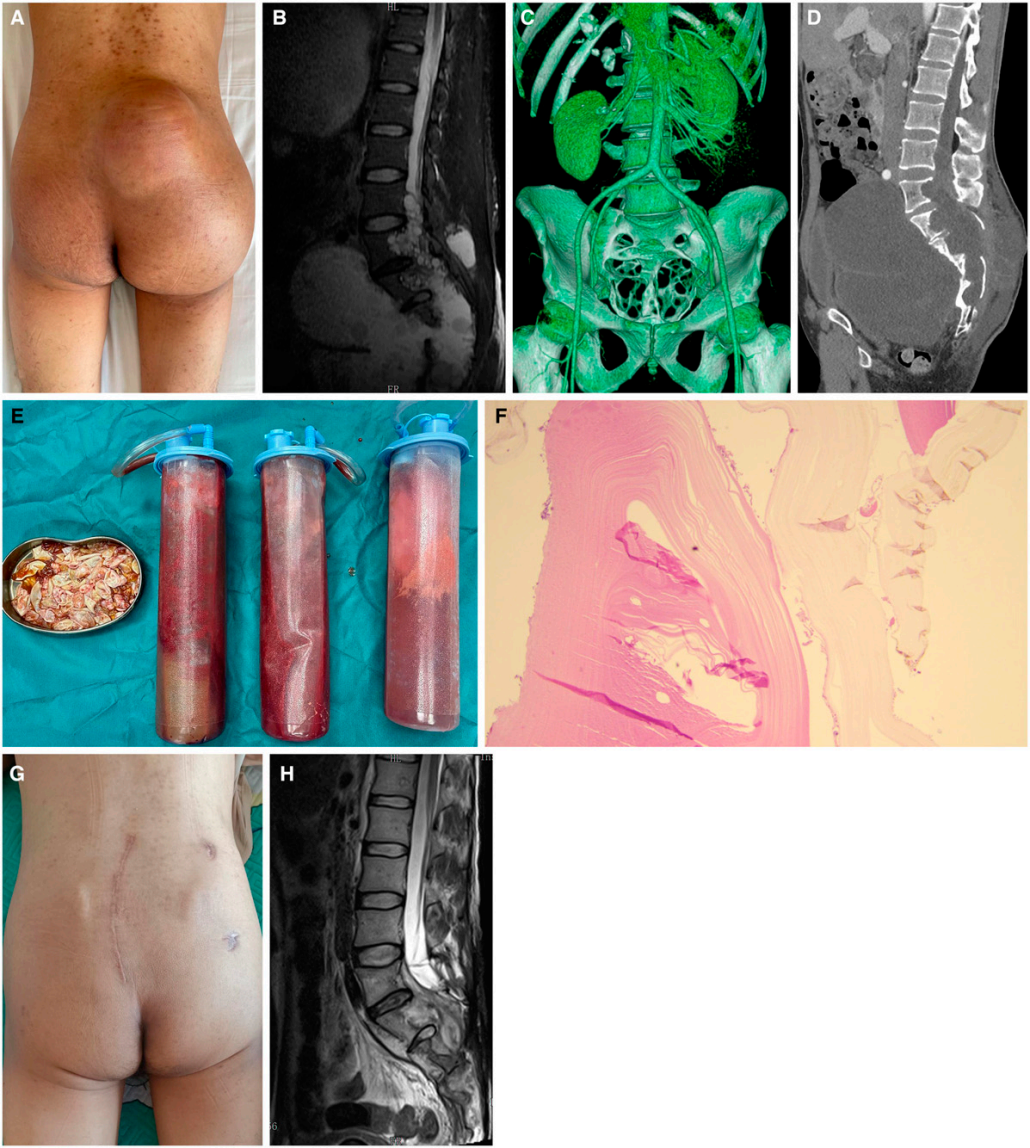
(**A**) Preoperative body photograph. (**B–D**) Preoperative magnetic resonance imaging (MRI) and computed tomography scans showed expansile multicystic lesions and destruction of the L5 vertebra, sacrum, and coccyx. (**E**) Removed lesions. (**F**) Lamellar membranous structure confirmed the diagnosis of cystic echinococcosis (×100). (**G** and **H**) Body photograph and MRI at 5-month follow-up.

A 32-year-old man presented to the outpatient department with 2 years of progressive right lower extremity weakness and numbness associated with bowel and urinary dysfunction. He had undergone surgical removal of hepatic echinococcosis about 28 years ago without anti-parasitic chemotherapy. Physical examination revealed a mass in the right buttock (Figure 1A), a lump in the lower abdomen, and impaired sensation and movement in the right lower extremity. Laboratory tests showed a normal white blood cell count and erythrocyte sedimentation rate and an elevated C-reactive protein of 15 mg/L (reference value, < 8 mg/L). Magnetic resonance imaging (MRI) showed multiple cystic lesions from the L4 vertebra to the sacrum. Cystic lesions were also found in pelvic cavity and liver (Figure 1B). Computed tomography imaging indicated that the L5 vertebra, sacrum, and coccyx were severely destroyed (Figure 1C and D).

The patient underwent surgical treatment (Figure 1E) and was prescribed albendazole (10 mg/kg/day). Histopathologic examination confirmed the diagnosis of cystic echinococcosis (Figure 1F). His symptoms significantly improved after surgery, and he returned to work 3 months later. At 5-month follow-up, MRI did not show any sign of recurrence (Figure 1G and H).

Echinococcosis is relatively common in the liver (∼70%) and lung (∼20–30%) but rarely affects the spine (∼0.2–1%).^1^ Spinal echinococcosis carries a high rate of morbidity and disability. Treatments for spinal echinococcosis include medication and surgery.^2^^,^^3^ Because most patients present with nerve compression symptoms, surgery is required in such instances.^1^ The invasive diffuse spread within bone leads to difficulty in radical resection of the echinococcus and a high relapse rate. Literature reported that patients treated by surgery together with chemotherapy were less likely to experience recurrence than those treated by only surgery.^1^ Therefore, after surgery, antihelmintic chemotherapy and close follow-up are encouraged for patients with spinal echinococcosis.^4^
